# Stimulation of Hepatic Ferritinophagy Mitigates *Irp2* Depletion-Induced Anemia

**DOI:** 10.3390/antiox12030566

**Published:** 2023-02-24

**Authors:** Yutong Liu, Yuxuan Li, Liu Yang, Jiaqi Shen, Hongting Zhao, Weichen Dong, Yanzhong Chang, Tong Qiao, Kuanyu Li

**Affiliations:** 1 Jiangsu Key Laboratory of Molecular Medicine, Medical School, Nanjing University, Nanjing 210093, China; 2 Department of Vascular Surgery, Affiliated Drum Tower Hospital, Medical School, Nanjing University, Nanjing 210093, China; 3 Department of Neurology, Affiliated Jinling Hospital, Medical School, Nanjing University, Nanjing 210002, China; 4 College of Life Science, Hebei Normal University, Shijiazhuang 050024, China

**Keywords:** IRP2, Hif2 inhibition, Fe-S clusters, ferritinophagy, microcytic anemia

## Abstract

Background: Iron regulatory proteins (IRPs) maintain cellular iron homeostasis. Due to aberrant tissue-iron distribution, *Irp2*-deficient mice suffer microcytic anemia and neurodegeneration, while iron overload occurs in the liver and intestine. We previously found that *Irp2* deficiency-induced Hif2 plays an important role in neurodegeneration. Methods: To test the role of Hif2 in *Irp2* deficiency-induced anemia, we used *Irp2* global knockout mice. Following Hif2 inhibition, routine blood tests, iron availability in bone marrow, histological assays, and biochemical analysis were performed to assess anemia improvement and tissue iron distribution. Results: We found that Hif2 inhibition improved anemia. The increased iron bioavailability for erythropoiesis was mainly derived from hepatic iron release, and secondly from enhanced intestinal absorption. We further demonstrate that nuclear receptor coactivator 4 (Ncoa4) was upregulated for iron release via the process of ferritinophagy. The released iron was utilized not only for intracellular Fe-S biogenesis but also for erythropoiesis after being exported from the liver to circulation. The hepatic iron export reduced hepcidin expression to further support iron absorption through the hepcidin-ferroportin axis to alleviate intestinal iron overload. Conclusion: Irp2 not only regulates cellular iron homeostasis but also tissue iron distribution by managing the involvement of Hif2-Ncoa4.

## 1. Introduction

Iron is essential for almost all living organisms. It functions as a cofactor in the form of Fe-S clusters or heme, or by itself, participating in numerous vital physiological processes, including mitochondrial respiration, oxygen transfer, DNA repair, and enzymatic catalysis [1,2,3]. While iron deficiency can lead to cognitive defects in children and anemia in adults, excess iron is also detrimental because it stimulates oxidative stress, subsequently causing tissue injury and disease [4]. As a result, it is imperative to maintain the body’s iron levels within an acceptable range.

To maintain iron homeostasis at the cellular level, iron regulatory proteins (IRP1 and IRP2) post-transcriptionally regulate the expression of iron-related proteins via the iron-responsive element (IRE) (see review in [5]). When iron is deficient, IRPs bind to the IRE in the 5’-untranslated region (5’-UTR) of L- and H-ferritin and ferroportin 1 (FPN1) mRNA to inhibit the translation of these genes to limit iron storage and efflux. Meanwhile, IRPs can stabilize mRNA by binding to the IRE in the 3’-UTR of divalent metal transporter 1 (DMT1) and transferrin receptor 1 (TfR1), which promotes iron uptake and ultimately alleviates iron deficiency [5,6]. In contrast, when iron is redundant, IRP1 binds to [4Fe-4S] clusters and loses its ability to bind IREs [7]; meanwhile, F-box and leucine-rich repeats protein 5 (FBXL5), a subunit of ubiquitin ligase complex, specifically recognizes IRP2 and promotes its degradation by gaining oxygen-responsive [2Fe-2S] clusters [8,9,10]. This, in turn, inhibits iron uptake, boosts iron storage, or exports excess iron.

Systemic deficiency of *Irp2* in mice results in microcytic anemia, erythropoietic protoporphyria [11], neurodegeneration [12], and diabetes [13,14], which are clinically manifested by phenotypes of patients with bi-allelic loss-of-function variants in *IREB2* [15]. *Irp2* depletion-induced microcytic anemia is not simply derived from global iron deficiency but is accompanied by iron overload in the liver and intestine [16]. This abnormal iron distribution might injure hepatocytes and enterocytes for liver metabolism and nutrient absorption.

Recently, we found that *Irp2*-null mutation causes downregulation of frataxin (Fxn) and IscU, two of the core components in the Fe-S cluster biogenesis machinery [17]. Consequently, mitochondrial dysfunction occurs, which shifts energy metabolism from oxidative phosphorylation (OXPHOS) to glycolysis in *Irp2*^−/−^ murine embryonic fibroblasts (MEFs) [18]. We further demonstrated that *Irp2* deficiency induces the expression of hypoxia-inducible factors Hif1α and Hif2α. Hif2, but not Hif1, suppresses mitochondrial Fe-S biosynthesis and OXPHOS in *Irp2*-deficient cells [18]. These results have been confirmed in *Irp2* knockout mice, showing that an increase in Hif2α switches energy metabolism from OXPHOS to glycolysis, and the inhibition of Hif2 by PT–2385, a selective Hif2 inhibitor, may alleviate the neurological disorder by improving mitochondrial function [19], which is accomplished by an increase of Fe-S biogenesis [18,19].

Mitochondria not only act as the powerhouse of the cell but also as Fe-S- and heme-houses. Two key enzymes in the second and last steps of heme synthesis (5’-aminolevulinate dehydratase (ALAD) in cytosol and ferrochelatase (FECH) in mitochondria, respectively) require Fe-S clusters as prosthetic groups to exert enzymatic activities [20,21]. We speculated that Hif2 inhibition might also improve *Irp2* deletion-induced microcytic anemia by enhancing Fe-S biogenesis. However, iron deficiency is induced by *Irp2* depletion in bone marrow [11,16]. Then, our question was where the erythroblasts in bone marrow would obtain enough iron.

In this study, we found that *Irp2* depletion-induced abnormal iron distribution was corrected by PT–2385 administration, indicated by a mitigation of iron accumulation in the liver and an increased iron content in hematopoietic tissue and serum. Further, we demonstrated that the iron release from ferritin in the liver is accomplished by nuclear receptor coactivator 4 (Ncoa4)-mediated ferritinophagy. Therefore, the symptoms of anemia were improved.

## 2. Materials and Methods

### 2.1. Animals and Tissue Collection

*Irp2* homozygous global knockout (*Irp2* KO) mice and WT mice in the C57BL/6J background were generated in our previous study [19]. Briefly, the *Irp2* KO and WT mice used in the experiment were the descendants of *Irp2* heterozygous knockout mice purchased from MMRRC at UC Davis (cat. no. 030490-MU, Davis, CA, USA). Mice were group-housed in standard housing conditions under a 12 h light-dark cycle at 25 °C. All experiments were approved by the Animal Investigation Ethics Committee of Nanjing University and were performed according to the Guidelines for the Care and Use of Laboratory Animals of the National Institutes of Health, USA.

The animals were weighed and euthanized after drug treatment. Blood was collected for subsequent routine blood and biochemical examinations. After saline perfusion, tissues including liver, kidney, and intestine were immediately frozen in liquid nitrogen or transferred to paraformaldehyde (4%) to be used as needed. Bone marrow was collected from the hind limbs of mice.

### 2.2. Drug Treatment

PT–2385 (MedChemExpress, Shanghai, China) dissolved in dimethylsulfoxide (DMSO) and diluted with saline was administered at 0.4 mg/kg bw by intraperitoneal injection qod for 1 month. The same DMSO volume was diluted with saline as the vehicle control. Six-month-old mice were randomly divided into four groups: WT mice with vehicle treatment (WT), WT mice with PT–2385 treatment (WT+PT–2385), *Irp2* KO mice with vehicle treatment (*Irp2* KO), and *Irp2* KO mice with PT–2385 treatment (*Irp2* KO+PT–2385).

### 2.3. Routine Blood Examinations

The red blood cell (RBC) count, hemoglobin (HGB) concentration, and mean corpuscular volume (MCV) (*n* = 8) were determined by a Mindray automatic hematology analyzer (BC-2800vet, Shenzhen, China).

### 2.4. Blood Biochemical Examinations

Blood was collected in heparinized tubes and centrifugated at 1200× *g* for 15 min at 4 °C. Serum samples were prepared for determination of alanine aminotransferase (ALT), aspartate aminotransferase (AST), and total bilirubin (TBil) by an auto-chemical analyzer (Beckman Coulter AU5421, Brea, CA, USA).

### 2.5. Western Blot Analysis

Mouse tissues were lysed by RIPA lysis buffer (Wuhan Servicebio Technology Co., Ltd., Wuhan, China) and homogenized by a KZ-II 2100 rpm High-Speed Tissue Homogenizer (Wuhan Servicebio Technology Co., Ltd., Wuhan, China). The protein concentration was quantified by Bradford buffer. Total proteins were prepared (20–40 µg/lane), run in SDS-PAGE gels at 100 V, transferred to nitrocellulose membranes, and incubated with primary and secondary antibodies for analysis.

The information for primary antibodies is as follows: anti-Hif2α (rabbit, 1:1000; cat# 109616), anti-ferritin light chain (rabbit, 1:1000; cat# 69090), anti-Ncoa4 (rabbit, 1:1000; cat# ab86707), and anti-Sdhb (rabbit, 1:2000; at# 178423) from Abcam (Cambridge, MA, USA). Anti-Ndufs1 (rabbit, 1:1000; cat# 12444-1-AP), anti-Uqcrfs1 (rabbit, 1:2000; cat# 1843-1-AP), anti-Atg5 (rabbit, 1:2000; cat# 10181-2-AP), anti-Glut1 (rabbit, 1:1000; cat# 21829-1-AP), anti-GAPDH (mouse, 1:5000; cat# 60004-1-Ig), and anti-Lamp1 (mouse, 1:1000′ cat#67300-1-Ig) from Proteintech (Chicago, IL, USA). Anti-LC3A/B (rabbit, 1:1000; cat# 4108) from Cell Signaling Technology (Danvers, MA, USA), anti-β-Actin (rabbit, 1:10,000; cat# AP0060) from Bioworld (Nanjing, China), anti-Fpn1 (mouse, 1:1000; cat# MTP11-A) from Alpha Diagnostic (San Antonio, TX, USA), anti-p62 (rabbit, 1:1000 cat# A7758) from ABclonal (Wuhan, China), and anti-Fxn, Iscu, Irp1, and Irp2 (polyclonal, self-made, raised from rabbits). All self-made antibodies were validated in previous studies [17,18]. The validation of the antibody against Fth is shown in Figure S1. The secondary antibodies were anti-rabbit IgG (HRP) (1:50,000; cat# 111-035-144) and anti-mouse IgG (HRP) (1:50,000; cat# 115-035-146) from Jackson ImmunoResearch Laboratories (West Grove, PA, USA). Quantification of the density of Western bands was performed with ImageJ software. Each experiment was repeated at least 3 times, independently.

### 2.6. Quantitative Real-Time PCR

Total RNA was isolated from tissues or cells using the RNA isolator Total RNA Extraction Reagent (Vazyme Biotech, Nanjing, China) and reverse-transcribed to cDNA by using HiScript^®^ III RT SuperMix for qPCR (+gDNA wiper) (Vazyme Biotech, Nanjing, China), according to the manufacturer’s instructions. RT-qPCR experiments were performed with ChamQ Universal SYBR qPCR Master Mix (Vazyme Biotech, Nanjing, China) in an Applied Biosystems ViiA™ 7 system. The following primers were used: for *Actin*, forward primer 5′-GCCACTGCCGCATCCTCTTC-3′ and reverse primer 5′-AGCCTCAGGGCATCGGAACC-3′; for *Epo*, forward primer 5′-AGTTGCCTTCTTGGGACTGA-3′ and reverse primer 5′-GCCACTCCTTCTGTGACTCC-3′; for *Hamp*, forward primer 5′-CTCCTGCTTCTCCTCCTTGC-3′ and reverse primer 5′-GCAATGTCTGCCCTGCTTTC-3′. *Actin* was used as a control.

### 2.7. Histological Staining

Peripheral blood smears were prepared by retro-orbital bleeding, then the smears were fixed in methanol for 10 min and stained with Wright-Giemsa stain (Wuhan Servicebio Technology Co., Ltd., Wuhan, China) at room temperature, following the manufacturer’s protocol.

For Prussian blue staining, tissue sections underwent dewaxing and hydration steps: dewaxing for 10 min in xylene twice, hydration for 5 min in 100–50% ethanol gradient buffers, rinsing for 5 min in running water at room temperature, and incubation in 1% potassium ferrocyanide in 0.12 M HCl for 30 min to 4 h. For liver slides and bone marrow smears, before being dehydrated by gradual ethyl alcohol solutions, sections were stained in Nuclear Fast Red Staining Solution (Wuhan Servicebio Technology Co., Ltd.) for 5–10 min; for small intestine slides, sections were color-rendered by 3, 3′-diaminobenzidin (DAB) (cat. no. D5905-1007AB; Sigma-Aldrich, Shanghai, China) for 40 min, and then dehydrated according to the same procedure as above.

Images were viewed through a biological microscope (BX43, Olympus, Tokyo, Japan) and captured using a digital camera.

### 2.8. Ferrozine Assays

The iron content of tissues was detected by ferrozine assays, as previously described [19]. Briefly, 11 μL of concentrated HCl (11.6 M) was added to 50 μL of tissue sample lysate or serum (250 μg total protein). The samples were heated at 95 °C for 20 min and centrifuged at the highest speed for 10 min. Then, 45 μL of supernatant was removed to a new tube. Next, 18 μL of ascorbate (75 mM), 18 μL of ferrozine (10 mM), and 36 μL of saturated ammonium acetate (NH_4_Ac) were sequentially added to each tube, with incubation for 2 min at room temperature between each step. The absorbance was read at 570 nm (BioTek ELx800, Shanghai, China).

### 2.9. ELISA

The serum EPO and interleukin-6 (IL-6) levels were quantified using specific ELISA kits according to the manufacturer’s instructions (Invitrogen, Waltham, MA, USA). 

### 2.10. Ferritin Iron Staining in Gels

In-gel ferritin iron staining was performed as described previously [22].Briefly, tissue samples of the lysate (200 μg total protein in 20 μL) were heated at 70 °C for 10 min and centrifuged at 12,000 rpm (~13,400× *g*) and 4 °C for 10 min. All supernatant was removed to a new tube and 4× loading buffer without β-mercaptoethanol (BME) and SDS was added. All samples were run in pre-cooled non-denaturing gels at 100 V for 4 h, at 4 °C. Finally, the gels were stained in Prussian blue staining buffer (1% potassium ferrocyanide in 0.12 M HCl) overnight. The data were collected by photography and quantified by ImageJ.

### 2.11. Statistical Analysis

All data are presented as mean ± SD. One-way ANOVA was performed using GraphPad Prism version 8.2.1 (La Jolla, CA, USA). *p*-value < 0.05 was considered significant.

## 3. Results

### 3.1. Hif2 Inhibition Ameliorates Anemia in Irp2 Knockout Mice

**Figure 1:**
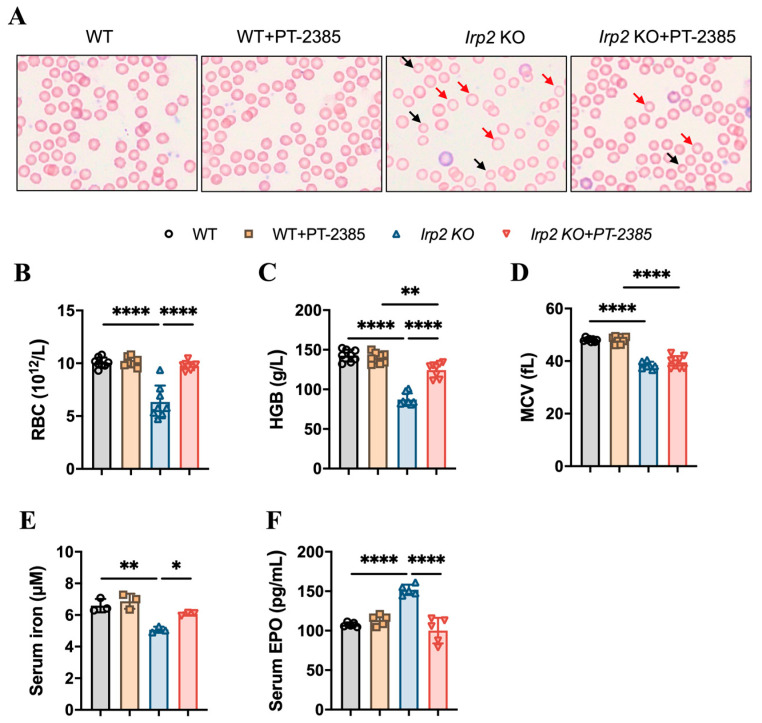
Hif2 inhibition by PT–2385 ameliorates anemia in *Irp2* KO mice. Six-month-old mice were divided into four groups: WT (DMSO as vehicle), WT+PT–2385 (0.4 mg/kg i.p. qod), *Irp2* KO (vehicle), and *Irp2* KO+PT–2385. (**A**) Improved hypochromic anemia by PT–2385 in *Irp2* KO mice on peripheral smear determined by Wright-Giemsa staining. Red arrows point to hypochromic red blood cells, black arrows point to small red blood cells. Routine blood examinations: (**B**) number of red blood cells (RBCs), (**C**) hemoglobin (HGB) concentration, and (**D**) mean corpuscular volume (MCV). (**E**) Serum iron content detected by ferrozine assays. (**F**) Serum EPO levels detected by enzyme-linked immunosorbent assays (ELISA). (**E**) *n* = 3, (**B**–**D**,**F**) *n* = 5–8. Results are shown as mean ± SD. One-way ANOVA was used for significance. * *p* < 0.05, ** *p* < 0.01, **** p < 0.0001.

To test the role of Hif2 in *Irp2* null-induced anemia in mice, six-month-old mice were randomly divided into four groups: wild-type (WT), WT+PT–2385, *Irp2* KO, and *Irp2* KO +PT–2385. PT–2385, as a selective Hif2 inhibitor, was administered (0.4 mg/kg bw i.p. qod) for 1 month. First, by monitoring the changes in body weight pre- and post-administration, we verified that intraperitoneal administration of PT–2385 did not affect mouse growth (Figure S2A). Physiological tolerance was assessed by measuring aspartate aminotransferase, alanine aminotransferase, and bilirubin levels before and after administration (Figure S2B–D), indicating that the dose of PT–2385 was within a safe range. Following that, we confirmed the presence of small hypochromic red blood cells in peripheral blood smears of *Irp2* KO mice and found the reverse phenotype following PT–2385 administration (Figure 1A). Other hematological indexes proved the significant rescue of *Irp2* KO-induced anemia by PT–2385 treatment in terms of red blood cell count and hemoglobin level (Figure 1B,C), with one exception: mean corpuscular volume (MCV) was not reversed by PT–2385 (Figure 1D), likely because one month of treatment was not enough time to improve all phenotypes.

It is known that Hif2 mediates the transcriptional activation of erythropoietin (*EPO*), a gene encoding a master hormone regulator of erythropoiesis, by binding 5’ hypoxia-responsive element (HRE), thereby contributing to the changes in tissue oxygen concentration for adaptive adjustment [23]. Our previous study [19] and others [11] have found that deletion of *Irp2* in mice increases Hif2α, leading to increased EPO, but this increase may be ineffective due to limited iron content in hematopoietic tissues. We wonder whether the improvement in anemia results from the increased iron availability when Hif2 is inhibited by PT–2385. To this end, we measured serum non-heme iron and EPO levels before and after PT–2385 administration. Consistent with previous studies [11], serum iron levels in *Irp2* KO mice were significantly lower than in WT mice, while serum EPO levels were higher. However, both changes remarkably returned following treatment with PT–2385 (Figure 1E,F).

### 3.2. Enhanced Fe-S Cluster Biogenesis by Hif2 Inhibition Rescues Iron Deficiency to Recover Hematopoietic Function in Irp2 Knockout Mice

**Figure 2:**
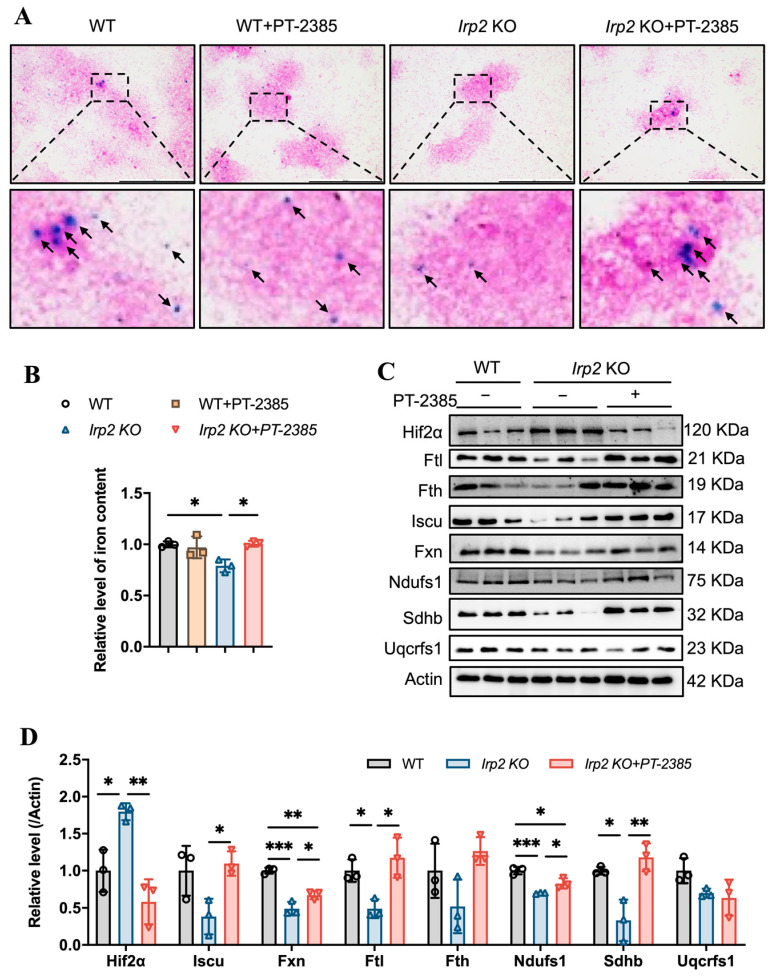
Hif2 inhibition increases available iron content and Fe-S cluster synthesis to maintain hematopoietic function in bone marrow. (**A**) Prussian blue staining of bone marrow smears. Arrows point to iron-stained positive areas. (**B**) Iron content in bone marrow determined by ferrozine assays. (**C**) Western blot analysis of expression of Hif2, iron-related proteins (Fth and Ftl), Fe-S cluster synthesis-related proteins (Fxn and Iscu), and respiratory chain-related proteins (Ndufs1, Sdhb, and Uqcrfs1) in bone marrow. Actin was used as an internal control. (**D**) Quantification of data in (**C**) to show significance; *n* = 3. Results are shown as mean ± SD. One-way ANOVA was used for significance. * *p* < 0.05, ** *p* < 0.01, *** *p* < 0.001.

Bone marrow is the vital hematopoietic tissue, and iron availability is one of the main factors affecting hematopoiesis. Previous studies have shown that *Irp2* KO-induced microcytic anemia in mice is derived from insufficient iron reserves in the bone marrow [11]. We expected that the therapeutic effect of PT–2385 on anemia would be proven due to the increasing iron availability in serum (Figure 1E). Therefore, we detected the iron content in bone marrow samples by ferrozine assays and in bone marrow smears by Prussian blue staining. Consistent with previous studies [11,16], *Irp2* deletion did cause iron deprivation in bone marrow, and PT–2385 treatment significantly reversed it to WT levels (Figure 2A,B). The biochemical data from Western blotting also provided evidence that Hif2α expression was significantly increased in *Irp2* KO mice but decreased after drug treatment, and that expression of ferritin H and L subunits (Fth and Ftl) increased with PT–2385 administration; in particular, Ftl significantly increased (Figure 2C, quantified in Figure 2D), indicating the rescued iron availability for erythropoiesis.

Our previous studies revealed that *Irp2* KO-induced functional iron deficiency downregulated the synthesis of mitochondrial Fe-S clusters [18], which are essential for mitochondrial heme biogenesis and mitochondrial function. Then, we asked whether mitochondria could use the available iron. Therefore, we detected the expression of proteins related to Fe-S cluster synthesis and mitochondrial complex in the bone marrow. The results showed that Fe-S cluster synthesis-related proteins Fxn and Iscu and mitochondrial complex I- and II-related subunits Ndufs1 and Sdhb more or less decreased, but significantly, in *Irp2* KO mice, except complex III subunit Uqcrfs1. However, the changed proteins recovered after PT–2385 administration (Figure 2C, quantified in Figure 2D), further in favor of the notion that *Irp2* KO-induced upregulation of Hif2 negatively modulates the mitochondrial OXPHOS [18,19].

**Figure 3:**
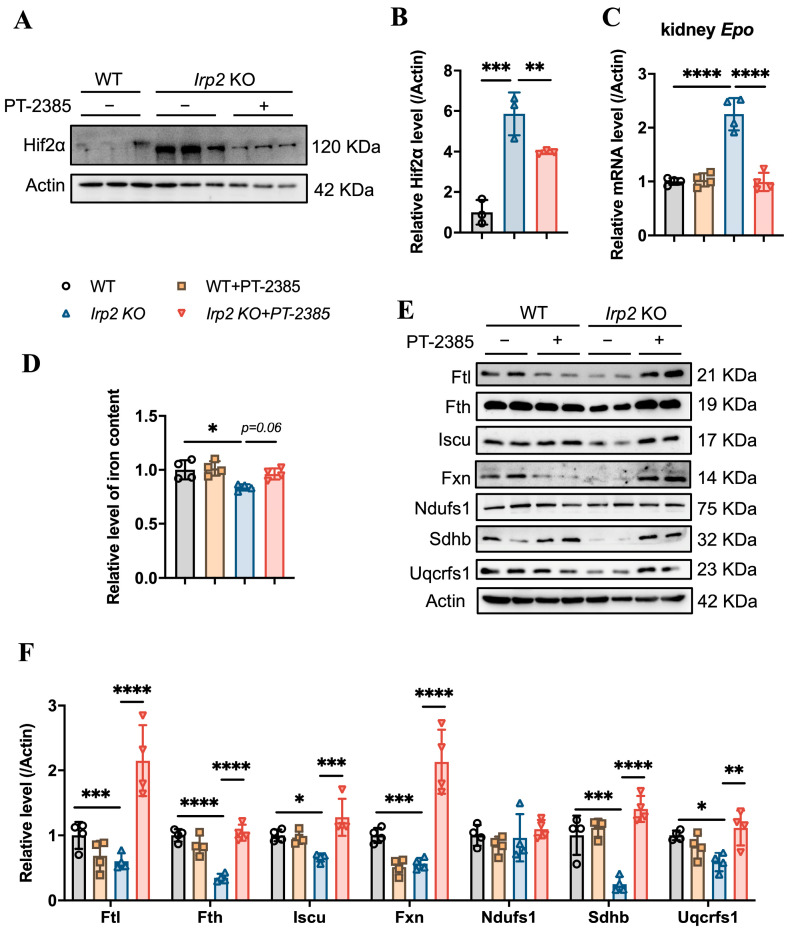
Hif2 inhibition reduces abnormally high EPO levels and rescues mitochondrial iron metabolism in kidneys. (**A**) Protein levels of Hif2α revealed by Western blot analysis. Actin was used as an internal control. (**B**) Quantification of Hif2α to show significance. (**C**) Relative mRNA levels of *Epo* in kidney, detected by quantitative real-time PCR. (**D**) Iron content in kidneys determined by ferrozine assays. (**E**) Western blot analysis of iron-related proteins (Fth and Ftl), Fe-S cluster synthesis-related proteins (Fxn and Iscu), and respiratory chain-related proteins (Ndufs1, Sdhb, and Uqcrfs1) in kidney. Actin was used as an internal control. (**F**) Quantification of data in (**E**) to show significance; *n* ≥ 3. Results are shown as mean ± SD. One-way ANOVA was used for significance. * *p* < 0.05, ** *p* < 0.01, *** *p* < 0.001, **** *p* < 0.0001.

On the other hand, inhibition of Hif2 might reduce the production of EPO, which is a direct target of Hif2 [23], to suppress hematopoiesis. EPO is primarily produced in the kidney rather than the bone marrow. Therefore, we detected the Hif2α protein and *Epo* mRNA levels in the kidneys, and the results confirm that the *Irp2* KO-induced upregulation of Hif2α (Figure 3A,B) and increased mRNA of *Epo* returned to the proper level after administration of PT–2385 (Figure 3C), accompanying an improvement in anemia in *Irp2* KO mice (Figure 1). Along with the mild increase of renal iron content (Figure 3D), the proteins related to Fe-S cluster biogenesis and mitochondrial function in the kidney recovered the most, comparable to WT, after administration (Figure 3E, quantified in Figure 3F), further supporting that mitochondrial dysfunction, more specifically Fe-S cluster insufficiency, is triggered by *Irp2* KO-induced upregulation of Hif2α [17,18].

### 3.3. PT–2385 Treatment Modulated Intestinal Iron Absorption and Hepatic Iron Release in Irp2 KO Mice

**Figure 4:**
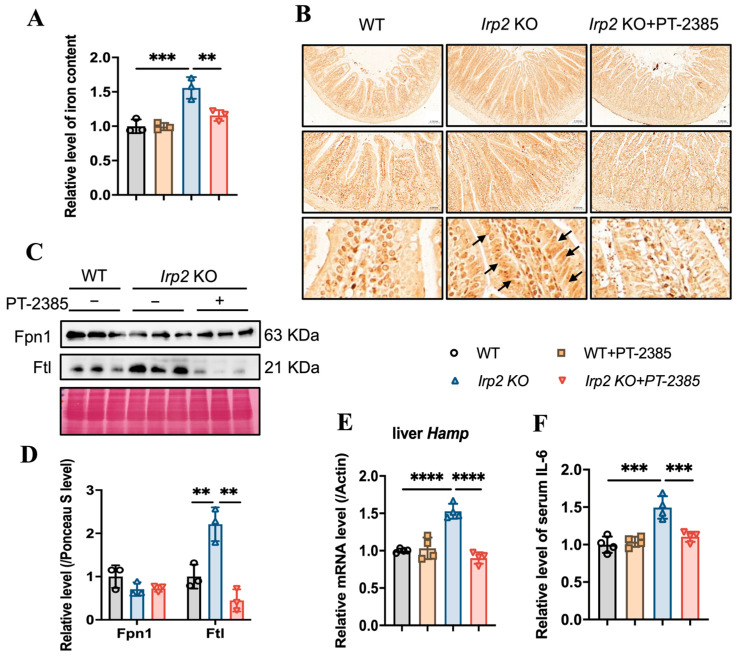
Abnormal iron accumulation is suppressed in the small intestine and hepcidin levels are downregulated in the liver of *Irp2* KO mice by PT–2385 treatment. (**A**) Iron content in the small intestine determined by ferrozine assays. (**B**) Representative images of DAB-enhanced Prussian blue staining for iron in the small intestine. Arrows indicate iron-stained epithelial cells. (**C**) Protein levels of Fpn1 and Ftl revealed by Western blot analysis. Ponceau S stain was used as an internal control. (**D**) Quantification of (**C**) to show significance. (**E**) Relative mRNA levels of Hepcidin in the liver, detected by RT-PCR. (**F**) Relative serum IL-6 levels, detected by ELISA. *n* ≥ 3. Results are shown as mean ± SD. One-way ANOVA was used for significance. ** *p* < 0.01, *** *p* < 0.001, **** *p* < 0.0001.

While iron deficiency has been found in bone marrow and kidneys in *Irp2* KO mice, iron overload has been found in the intestine and liver [16]. We wondered whether and how the available iron came from the intestine for more absorption or from the liver for more iron release. Based on this question, we first determined the changes in iron content in the small intestine. The results showed that iron accumulation was inhibited in the small intestine of *Irp2* KO mice after PT–2385 treatment, as demonstrated by decreased total iron content (Figure 4A), fewer iron-stained positive areas (Figure 4B), and reduced ferritin expression (Figure 4C). This inhibition was correlated with mildly increased intestinal Fpn1 (Figure 4C, quantified in Figure 4D) and remarkably decreased liver hepcidin (*Hamp*) expression (Figure 4E). Liver is the primary site of synthesis and secretion of hepcidin. Its expression is regulated by inflammation and iron, allowing us to detect the expression of inflammatory factor IL-6. The ELISA results showed an increase in the level of serum IL-6 in *Irp2* KO mice, which decreased after PT–2385 administration (Figure 4F). Other inflammatory markers (interleukin-1β and C-reactive protein) were not found to be changed in *Irp2* KO mice.

**Figure 5:**
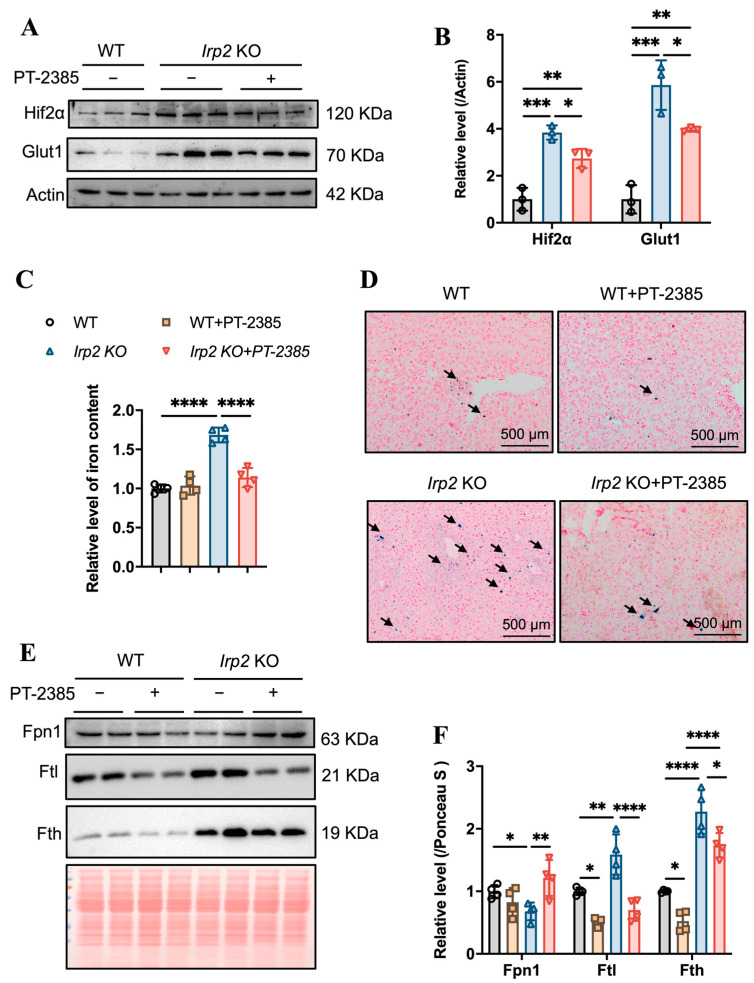
PT–2385 treatment inhibits iron accumulation in the liver. (**A**) Protein levels of Hif2α and Glut1 revealed by Western blot analysis. Actin was used as internal control. (**B**) Quantification of (**A**) to show significance. (**C**) Ferrozine-based iron content in the liver. (**D**) Prussian blue staining of liver sections. Arrows indicate stained iron. (**E**) Iron-related proteins (Fpn1, Fth, and Ftl) detected by Western blot analysis. Ponceau S stain was used as an internal control. (**F**) Quantification of (**E**) to show significance. *n* ≥ 3. Results are shown as mean ± SD. One-way ANOVA was used for significance. * *p* < 0.05, ** *p* < 0.01, *** *p* < 0.001, **** *p* < 0.0001.

Hepcidin regulates iron efflux by mediating Fpn1 endocytosis and ensuring that hepatic iron can be mobilized to maintain iron homeostasis [24]. We expected that the iron accumulation would be alleviated due to the diminished *Hamp*. First, we detected the effect of PT–2385 on Hif2α expression. As in other tissues, Hif2α was induced by *irp*2 ablation and reduced by PT–2385 treatment, and the Hif2α-targeted *Glut*1 gene showed a similar pattern (Figure 5A,B). Indeed, we found that PT–2385 could effectively reduce the accumulation of hepatic iron caused by *Irp2* deletion, verified by ferrozine assays and Prussian blue iron staining (Figure 5C,D). Biochemically, iron-related proteins Fpn1, Fth, and Ftl all changed to present reduced iron accumulation (Figure 5E,F).

Collectively, these results indicate that PT–2385 administration could enhance iron absorption in the gut and iron release from the liver to rebuild iron homeostasis and proper hematopoiesis in *Irp2* KO mice.

### 3.4. Hif2 Inhibition Reconstitutes Tissue Iron Homeostasis by Initiating Ncoa4-Mediated Ferritinophagy in Liver

We wondered what mediates the remobilization of iron from the liver after Hif2 inhibition. In addition to maintaining systemic iron homeostasis through the hepcidin-FPN1 axis and cellular iron homeostasis through the IRP-IRE system, another regulatory strategy has been revealed in some tissues or cells, such as red blood cells and macrophages, as Ncoa4 mediates ferritinophagy, a process that maintains intracellular iron homeostasis to sustain erythropoiesis [25,26].

**Figure 6:**
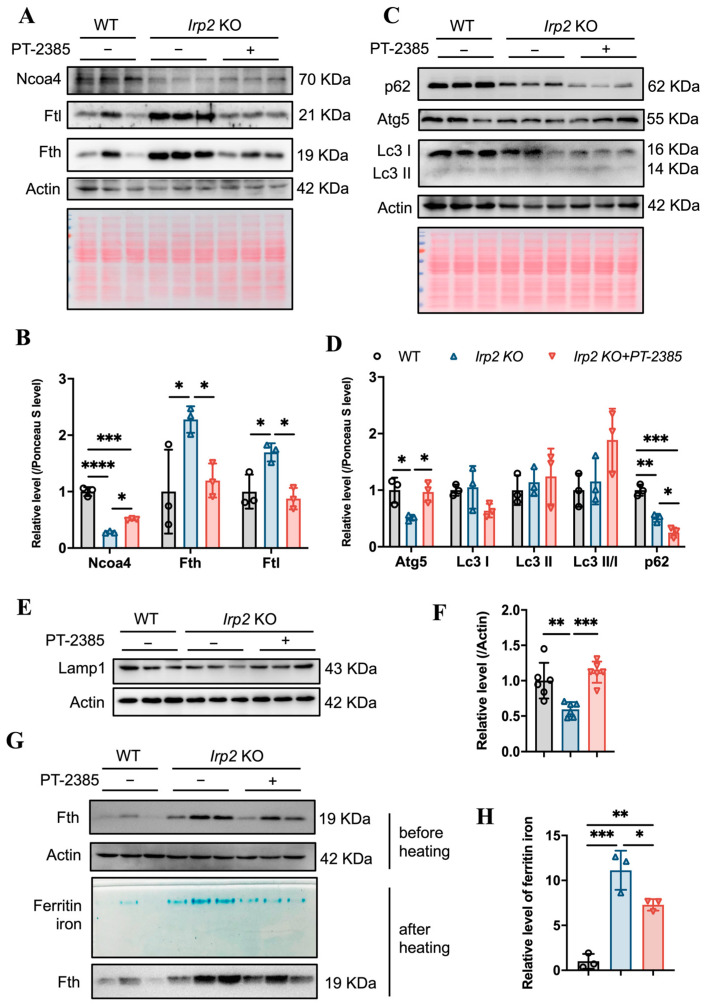
Hif2 inhibition stimulates Ncoa4-mediated ferritinophagy for iron release from ferritin in *Irp2* KO liver. (**A**) Expression of Ncoa4 and iron-related proteins (Fth and Ftl) after PT–2385 treatment in liver of *Irp2* KO and WT mice. Actin and Ponceau S stain were used as internal controls. (**B**) Quantification of (**A**) to show significance. (**C**) Expression of autophagy-related proteins (Atg5, Lc3, and p62). Actin and Ponceau S stain were used as internal controls. (**D**) Quantification of (**C**) to show significance of Atg5, Lc3, and p62 changes. (**E**) Lamp1 expression. Actin was used as an internal control. (**F**) Quantification of Lamp1 to show significance. (**G**,**H**) Result of ferritin iron staining in gels. *n* ≥ 3. Results are shown as mean ± SD. One-way ANOVA was used for significance. * *p* < 0.05, ** *p* < 0.01, *** *p* < 0.001, **** *p* < 0.0001.

Considering that Ncoa4 is a critical cargo receptor for autophagic degradation of ferritin and the subsequent release of iron, we speculated that PT–2385 treatment could induce Ncoa4-mediated ferritinophagy based on the decline in ferritin. Therefore, we examined the expression level of Ncoa4. It turned out that Ncoa4 expression increased (Figure 6A, quantified in Figure 6B), in agreement with the mitigated iron accumulation and lower ferritin levels in the liver, comparable to WT mice (Figure 5C,E). Subsequently, we examined the autophagic elongation component Atg5, the autophagic cargo selection component Lc3, and the classical autophagy receptor p62. Atg5 forms a constitutive complex with Atg12 to promote the transfer of Lc3-I to phosphatidylethanolamine (PE). Lc3-I is then lipidated and converted to Lc3-II by ATG4 during autophagy activation, and p62 engages with autophagic substrates and carries them to autophagosomes for degradation (see review in [27]). As a result, the Lc3-II/Lc3-I ratio and decreased p62 levels are widely used as markers of autophagy activation. Consistent with the increased Ncoa4 levels, the expression of Atg5 significantly increased, the Lc3-II/Lc3-I ratio slightly increased, and p62 levels decreased in *Irp2* KO mice after treatment with PT–2385 compared with the vehicle group (Figure 6C,D), indicating an increase in the autophagic process in the liver. Autophagy is a major intracellular system that derives its degradative abilities from the lysosome. We then detected the lysosomal-associated membrane protein 1 (Lamp1). The results showed that Lamp1 protein levels were significantly lower in *Irp2* KO mice, and were increased after treatment, comparable to WT levels (Figure 6E,F). This suggests that *Irp2* deletion may have a direct effect on lysosomal biogenesis and function and, ultimately, autophagic flow, explaining why ferritinophagy efficiency is reduced in irp2 KO mice despite a decrease in p62 levels. In addition, to measure loaded iron in ferritin, we stained the iron by in-gel assays [22] and found significantly less iron in the liver after PT–2385 treatment (Figure 6G,H), correlating with the ferritin levels (Figure 6A).

In summary, Hif2 inhibition by PT–2385 stimulated the release of iron from hepatic ferritin storage by commencing Ncoa4-mediated ferritinophagy, and ultimately corrected the *Irp2* KO-induced aberrant iron distribution in tissues for appropriate erythropoiesis.

## 4. Discussion

**Figure 7:**
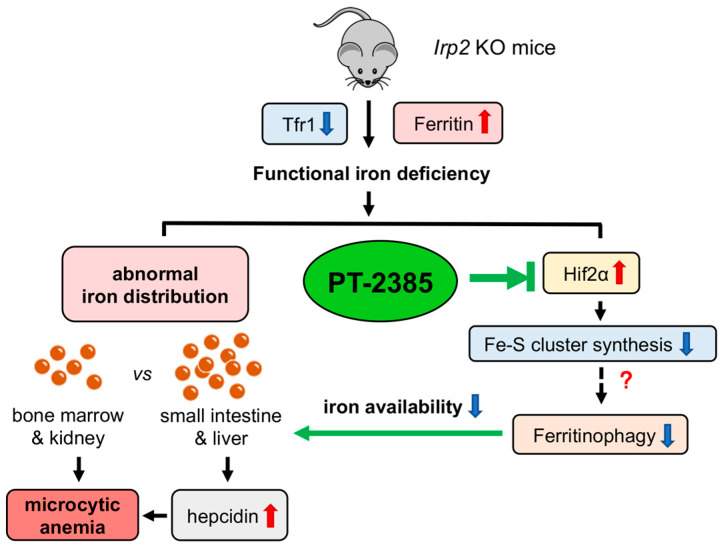
Working model of the role of *Irp2* KO-induced upregulation of Hif2 in microcytic anemia. *Irp2* deficiency causes functional iron deprivation by reducing cellular iron uptake (downregulated transferrin receptor 1, Tfr1) and increasing cellular iron storage capacity (upregulated ferritin), eventually leading to the development of microcytic anemia symptoms in *Irp2* KO mice. Iron accumulation was observed in the intestine and liver, but iron deficiency was seen in erythropoiesis-related organs such as bone marrow and kidney. At the molecular level, *Irp2* deficiency increases Hif2α expression, interferes with Fe-S cluster synthesis, and may affect ferritinophagy, resulting in decreased iron availability. Furthermore, elevated levels of hepcidin secreted by the liver aggravate the abnormal iron distribution. Hif2α inhibition by PT–2385 alleviated symptoms of anemia in *Irp2* KO mice by increasing ferritinophagy levels.

Here, we observed that inhibition of Hif2 by PT–2385 could alleviate *Irp2* depletion-induced anemia (Figure 7). Mechanically, it was revealed that upregulation of Ncoa4 initiated ferritinophagy in the liver to release iron into the serum for erythropoiesis in bone marrow. Concurrent with the reduced liver iron, liver *Hamp* expression decreased, which systemically affected iron absorption and recycling. Liver Fpn1 was found to be significantly upregulated though intestinal Fpn1 only slightly, and it is expected that the efficiency of iron transportation was increased in the intestine and liver due to the reduced hepcidin after PT–2385 administration. Therefore, anemia is cured, and aberrant iron distribution is corrected (Figure 7).

Dietary iron acquisition in mammals is mediated by intestinal absorption. In the proximal gut, ferric iron (Fe^3+^) is reduced to ferrous iron (Fe^2+^) by duodenal cytochrome b (Dcytb), a ferric reductase [28], and then transported across the apical membrane by divalent metal transporter 1 (Dmt1) [29]. According to the body’s need for iron, iron can be either kept in ferritin or exported via the only known iron exporter, Fpn1, over the basolateral membrane and into the circulation [30]. Three major intestinal proteins (Dcytb, Dmt1, and Fpn1) have been identified as direct targets of HIF2α, and oral administration of HIF2α inhibitors (1,3-diaminopropane, DAP, and reuterin) significantly suppressed iron uptake via the intestine, implying that HIF2α is required to maintain intestinal iron absorption [31]. Interestingly, in our study, when *Irp2* KO mice were administered the HIF2α inhibitor PT–2385 intraperitoneally, there was no significant change in body weight. In addition, no significant change of intestinal Fpn1 expression was observed, while the excessive iron accumulation in the small intestine was significantly alleviated after Hif2α inhibition. This discrepancy suggests efficient iron transport via Fpn1 over the basolateral membrane and, to some extent, intraperitoneally administrated PT–2385 being bypassed from the gut. How *Irp*2 ablation induces Hif2α upregulation in the liver even though iron accumulation was observed there remains a question. This phenomenon was observed previously in a study showing that the Hif2α protein level remained constantly high even under iron repletion conditions in *Irp*2-ablated cells [18]. Probably, the iron- and oxygen-dependent degradation process is disturbed when *Irp*2 is depleted. These results also support the finding that besides IRP1 [32,33,34], IRP2 also regulates Hif2α via an unknown mechanism.

As a primary circulating regulator of iron, hepcidin regulates three significant iron fluxes into plasma: dietary iron absorption in the intestine, iron recycling by macrophage phagocytosis, and iron mobilization from hepatocytes (see review in [35]). A recent study showed that when erythropoiesis intensifies, EPO increases erythroferrone (ERFE) production in the bone marrow and spleen erythroblasts in a JAK2/STAT5-dependent manner, and ERFE is secreted into the bloodstream and acts directly on the liver to suppress hepcidin [36]. Even though EPO levels are significantly increased in *Irp2* KO mice, hepcidin is not suppressed. On the contrary, hepcidin is expressed more than in wild-type mice, suggesting that the increase in EPO is invalid [11] and is the consequence of iron deficiency in the bone marrow. In addition, inflammatory signals in the body may induce hepcidin expression. For instance, interleukin-6 upregulates hepcidin expression in vivo through the IL-6/JAK2/STAT3 pathway [37], and other cytokines, such as IL-1β, that rely on BMP/SMAD signaling also increase hepcidin expression [38]. In this study and our previous study [19], we found an increase in serum IL-6, not IL-1β, in *Irp2* KO mice and a decrease in IL-6 after PT–2385 administration. The decreased Fpn1 in the intestine and liver of *Irp2* KO mice and the increase in the liver after Hif2 inhibition confirm that hepcidin is a master regulator of Fpn1, though *Fpn1* is a member of the Hif2 regulons.

Iron mobilization from hepatocytes through Fpn1, we think, plays a very important role in correcting the aberrant iron distribution in *Irp2* KO mice. Iron, if not utilized or exported, is stored in ferritin nanocages consisting of 24 subunits of FTH1 and FTL per cage. The cage is able to hold up to 4500 iron atoms [1]. What causes iron to release from ferritin after Hif2 inhibition? Ferritin is regulated at least at three levels, transcriptionally by NFκB [39], post-transcriptionally by the IRE-IRP system [40], and post-translationally by Ncoa4-mediated ferritinophagy [41]. In this study, we found a decrease in the level of ferritin protein, not mRNA, in *Irp2* KO mice after PT–2385 administration, which led us to hypothesize that PT–2385 treatment activates Ncoa4-mediated ferritinophagy. Ncoa4 interacts with the ferritin heavy chain and mediates ferritin transport to the lysosome via the autophagosome for degradation, which is essential for maintaining iron homeostasis, especially in erythrocytes [26,42]. In agreement with these findings, PT–2385 treatment increased Ncoa4 expression and autophagic flux in the liver, which led to a massive efflux of hepatic stored iron via Fpn1 and, consequently, to circulation for iron redistribution and erythropoiesis in bone marrow. Though Ncoa4 is regulated by Hif at the transcriptional level and mediates the mobilization of murine hepatic iron stores after blood loss [43], we found that inhibition of Hif2 increased Ncoa4 expression, suggesting post-transcriptional regulation of Ncoa4. Very recently, Ncoa4 has been demonstrated to be selectively targeted for the management of iron overload disorders [44]. This explains our finding that increased Ncoa4 alleviated liver iron overload to improve systemic iron homeostasis. In addition, Ncoa4 has been revealed to be regulated by iron-dependent HERC2-mediated proteolysis [42]. We wonder if a dependence on Fe-S clusters, besides iron, is also possible, because Hif2 inhibition by PT–2385 shifts cellular energy metabolism from glycolysis to oxidative phosphorylation by enhancing Fe-S cluster biogenesis and mitochondrial function to improve *Irp2* KO-induced neurodegeneration [18,19]. This possibility needs to be further investigated.

Overall, we validated the role of Hif2 in *Irp2* KO-induced anemia. Combining our findings and others’ previous findings, we have highlighted that Hif2α-Ncoa4 is a complex axis that contributes to iron metabolic disorders, including neurodegeneration, iron-overload disorder, and anemia.

## Data Availability

Data is contained within the article and supplementary material.

## Supplementary Materials

The following supporting information can be downloaded at https://www.mdpi.com/article/10.3390/antiox12030566/s1: Figure S1. The validation of self-made antibody against Fth. Figure S2. Body weight and blood biochemical indices.
