# Supplementary material for: Stimulation of Hepatic Ferritinophagy Mitigates Irp2 Depletion-Induced Anemia
Source: Antioxidants (Basel). 2023 Feb 24;12(3):566. doi: 10.3390/antiox12030566 (PMC10044941; doi:10.3390/antiox12030566)
Supplement: Supplementary file 1 [file antioxidants-12-00566-s001.zip › antioxidants-2192265-supplementary.pdf]

Supplementary materials

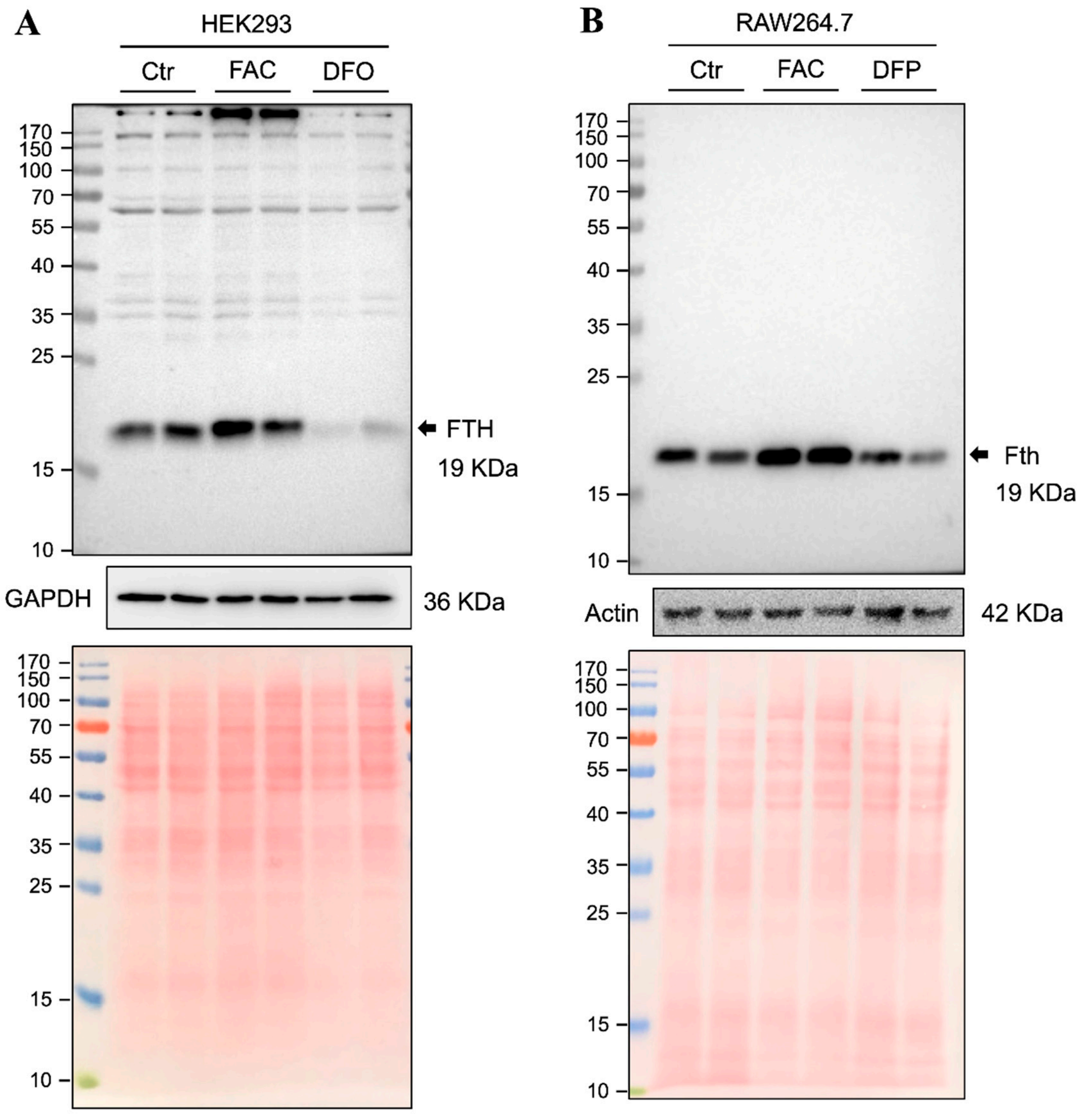

Figure S1. The validation of self-made antibody against Fth. Evaluation of Fth antibody with Western blot analysis in HEK293 cells (A) and RAW264.7 cells (B) after treatment with 200 µM FAC or 100 µM DFO for 24 h. Top panel: regular immunoblotting anti-Fth; middle panel: anti-GAPDH (A) or anti-β-Actin (B) used as an internal control; bottom panel: Ponceau S staining.

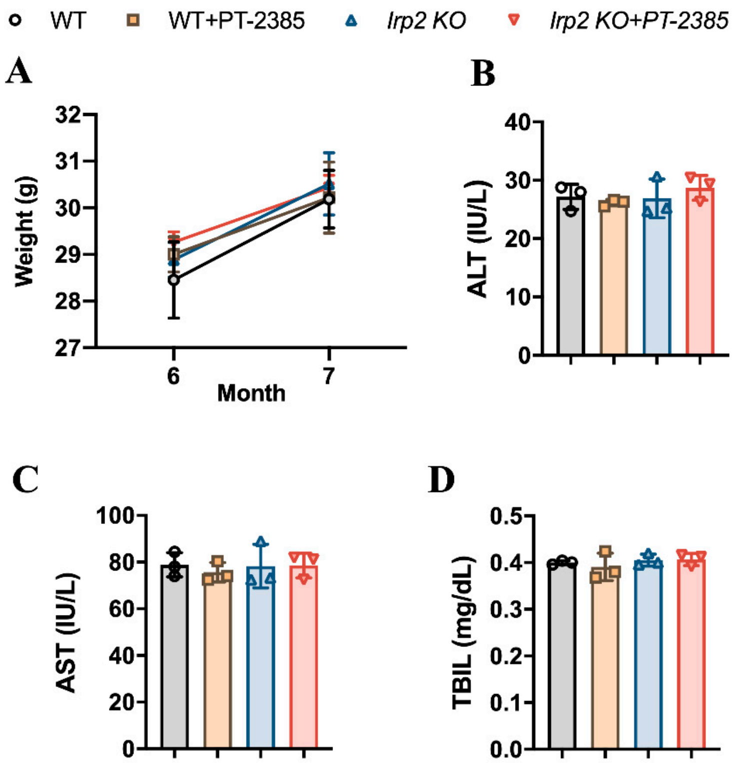

Figure S2. Body weight and blood biochemical indices. (A) Changes in body weight of mice during drug administration. Blood biochemical parameters: (B) alanine aminotransferase (ALT), (C) aspartate aminotransferase (AST), (D)and total bilirubin (TBil). (A) n=6, (B-D) n=3. Results are shown as the mean ± SD. One-way ANOVA was used for significance.
